# DORA on steroids

**DOI:** 10.1038/s44319-024-00068-y

**Published:** 2024-01-25

**Authors:** Howy Jacobs

**Affiliations:** Tampere University, Tampere, Finland

**Keywords:** Science Policy & Publishing

## Abstract

A much better system is needed to recognize the contributions of individual authors to multi-authored works.

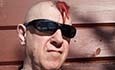

Although raw bibliometrics—notably journal impact factor—are now widely frowned upon as measures of scientific achievement, we are still faced with the task of how to apportion value to the authorship of scientific papers; in particular, to the contributions of individual authors, when considering candidates for grants, fellowships or jobs. The San Francisco Declaration on Research Assessment, DORA (https://sfdora.org/read/) enjoined evaluators to consider the true significance of published works in the field of study, rather than the name or reputation of the journals in which they were published. But it only scratched the surface of proper attribution.

Back in the day, it was quite common for a doctoral student to publish their work as a single-author paper without their advisor as a co-author. An acknowledgment of their support or mentorship was often considered sufficient recognition. Contrast that with the increasing trend, especially in the biomedical sciences, for ‘high-value’ papers to have tens of authors, whose individual contributions are impossible to discern from reading the paper alone. Just to take the most basic example, how can one apportion credit between the person who conceived the research project, the person who conducted the laboratory work, and the person who actually wrote the paper, who might be three different people. Or a supervisor who just threw in a few crucially important suggestions as the project unfolded.

Even if we had a way of distinguishing these contributions quantitatively, adequately attributing works involving more than one PI, collaborators on different continents, teams of researchers deploying diverse technical skills, or even just two graduate students, would still be highly problematic. The International Committee of Medical Journal Editors has set out general principles of attribution (https://www.icmje.org/recommendations/) but, in my opinion, these do not adequately address the main difficulty, of deciding how to value each author’s work.

To get around the problem, journals have recently adopted a variety of additions. It is now common practice to list the types of contribution each author made, according to a standard classification, such as the CrediT (Contributor Roles Taxonomy) system (https://credit.niso.org/). Unfortunately, its categories are still rather generic and overlapping—such as conceptualization, methodology, investigation and so on—and are often inapplicable or unclear in relation to discipline-specific papers. Some journals now invite an optional one-line specification to be added, or a rider indicating whether the contribution was ‘major’ or ‘supporting’, or even a numerical % contribution (Boyer S et al, 2017). EMBO Press has gone a step further, enabling individual figures in a paper to be attributed to specific authors. However, even these qualifiers don’t constitute a full measure of what each individual author brought to the work, neither in practical nor intellectual terms. They are also potentially disputatious, at least up until the final version of a paper is agreed and accepted, sometimes following many rounds of revision, and incorporating additional experimental data.

In addition, we will still be confronted with questions such as how can we compare 9 months of round-the-clock work in the lab that produced the data for one supplementary figure, to a single momentary flash of insight into what it all meant? And is second authorship on a three-author paper published in a ‘minor’ society journal worth more or less than being fifteenth author on a thirty-author paper published in a leading journal, and which is cited hundreds of times for a year or two before being forgotten about because the field has moved on?

The only answer I can give is that we need to expand on the DORA concept. In other words, that we need to consider the quality and impact of each author’s contribution to every published work, if we are fairly to evaluate that person—or their department or institute—in a competitive or comparative exercise. In my view, this requires two things that are missing from the current publishing and research assessment systems. First, we need a detailed, free-form statement from every author of the accepted manuscript, stating precisely what they did that justified their authorship. The set of such statements should be vetted and certified by the corresponding author, who must also take responsibility for confirming that it has been seen and approved by all authors, as already applies to the actual text of the article. These statements must be accessible to all readers. The task will be onerous and laborious but, otherwise, evaluators are just left guessing.

Second, noting that evaluators must spend a lot of time digging into these details if we are to apportion due credit for published work, we must find a way of rewarding those who devote time, effort and expertise to the task. If these duties are considered part of the evaluator’s job description, their institution needs to take them into account in awarding tenure, renewal, promotion or performance-related pay increases. And not merely state that it does so, but actually document how much weight it places on each of these activities and how it evaluates them. And be prepared to show how it did so in individual cases, if raised by a regulator, tribunal or labour union.

Alternatively, if review tasks are considered to be outwith the employee’s contractual obligations, academics should be financially remunerated for their time and specialist knowledge in performing them, in the way a lawyer, dentist or motor mechanic are paid for their time. This will raise the costs associated with publishing and research, and the costs will need to be passed on or recouped in some way. So be it.

All this sounds horribly bureaucratic, expensive and time-consuming. But without it we may as well decide promotions and so on on the basis of a coin toss or astrological chart (many authors and applicants suspect that such systems are already in use to triage their submissions). We could perhaps make a start by abolishing the linear order of authors presented on the title page of every scientific paper. If the information on who did what is included, we just need a footnote listing authors alphabetically by surname, or displayed in a randomly permuted order. Some disciplines already use alphabetical order for author lists—it’s surely time to make this universal, at least for papers with more than 3 authors, and with full descriptions of what they each actually did embedded under their names.
